# How multisensory perception promotes purchase intent in the context of clothing e-customisation

**DOI:** 10.3389/fpsyg.2022.1039875

**Published:** 2022-12-23

**Authors:** Pei Li, Xiangmei Guo, Chunmao Wu, Charles Spence

**Affiliations:** ^1^Department of Fashion Design and Engineering, School of Textiles and Fashion, Shanghai University of Engineering Science, Shanghai, China; ^2^Department of Product Design, College of Fashion and Design, Donghua University, Shanghai, China; ^3^Crossmodal Research Laboratory, Department of Experimental Psychology, University of Oxford, Oxford, United Kingdom

**Keywords:** multisensory perception, e-customisation, perceived arousal, perceived dominance, purchase intent

## Abstract

With the continuing development of internet technologies, an increasing number of consumers want to customise the products they buy online. In order to explore the relationship between perception and purchase intent, a conceptual framework was developed that was based on the link between multisensory perception, positive emotions, and purchase intent in fashion e-customisation marketing. We discuss the outcomes derived from consumers’ experiences in fashion e-customisation and analyse the relationships between variables. Questionnaires were used to collect data for this quantitative study (*n* = 398 participants). The data was analysed using factor analysis, correlation analysis, and regression analysis. The findings contribute to the field of clothing e-customisation by identifying the effects of visual perception, haptic imagery, and auditory stimulation on arousal, and purchase intent. Visual perception and haptic imagery exerted a positive influence over dominance. We also identify the effects of arousal and dominance on purchase intent, and assess the mediating effects of these variables on visual perception, haptic mental imagery, and purchase intent. The results highlight how fashion e-customisation marketing strategies can be adopted by managers in order to increase positive emotions and how multisensory perception can potentially be used to influence consumers’ purchase behaviour.

## 1. Introduction

E-customisation is an effective approach to providing products that meet consumers’ personalized needs (Park et al., 2013; Zhang et al., 2020), with consumers increasingly being motivated to engage in product customisation (Wang et al., 2022). Apparel customisation involves developing a customised design, selecting sizes, styles, fabrics, assembling, and personal adjustment (Cho and Fiorito, 2009). Consumers can be integrated into the clothing co-design and customisation process (Li and Liu, 2020; Shi et al., 2022). Using a 3D body scanner, the customised garment can be adopted by disabled people who are afflicted with scoliosis, that designers and consumers can collaborate to work easily (Hong et al., 2017). Online shopping and fashion e-customisation systems may also influence the consumers’ perceptions, behaviour, and emotions while browsing/purchasing (Li et al., 2020). Within a retail atmosphere, congruent multisensory cues (e.g., auditory, olfactory, and visual) have been shown to exert a positive effect on consumers’ emotions and purchase behaviour (Helmefalk and Hultén, 2017). In fact, many different stimuli affect the perception, emotional state, and behaviour of consumers (Spence and Gallace, 2011; Spence et al., 2014). Researchers have found that multisensory congruence impacts consumers’ purchase intentions and the amount of time that the latter spend shopping (Helmefalk and Berndt, 2018). For example, clothing is presented in videos *via* the smart Tao of Taobao and JD.com (platforms showing clothing videos in China), thus enriching the consumer’s audiovisual experience.

Meanwhile, consumers are involved in the online design process, choosing product attributes, and selecting the options from the interface and create a new product that may also reduce the cost of design (Zhang et al., 2020). Researchers also found that service excellence, clear navigation, and aesthetic appeal make it easy for consumers to get accurate information in the context of clothing e-customisation (Lang et al., 2021). The clothing e-customisation system provides product information concerning certain brands, such as Cotte Yolan, NYB, Vans, Uniqlo, and Nike ID, *via* consumers’ computers and mobile applications. Woolley et al. (2021) developed a system for the mass customization of food supply and optimized the recipe selection so as to reduce food waste. Furthermore, multisensory stimuli can be adopted by e-retailers in order to present the character of products in interfaces and help to differentiate them from their competitors. For retailers, sensory perception is directly related to consumers’ decision-making (Helmefalk and Hultén, 2017). E-retailers use visual images and auditory information to influence sensory perception in online shopping (Petit et al., 2019). Advertisements that provide attractive pictures and videos to consumers that may help them to get connection with the products in online shopping (Charoensereechai et al., 2022). Both pictorial information and verbal descriptions of the fabric can enable the consumer to get indirect haptic cues that may facilitate their purchase decision (Rodrigues et al., 2017). At the same time, however, an excess of visual stimuli (what some have referred to as ‘sensory overload’; Malhotra, 1984) has sometimes been presented in online shopping, leading to complex emotions and behaviour in consumers.

In the context of e-customisation, the consumers’ intentions and behaviour are driven not only by their perception but also by their feelings and emotions. Indeed, researchers have found that emotions are a vital concept in the field of consumer behaviour and decision-making (Lerner et al., 2015; Kautish et al., 2021). Both positive and negative emotions contribute to the pleasure and happiness of consumers, and these are often stimulated by innovative technology (Valor et al., 2022). For example, people rate chocolate as having a softer texture and are more willing-to-purchase it, when listening to music that is positive rather than negative (Reinoso-Carvalho et al., 2020b). Product customisation involves the consumer’s perception and experiences which, in turn, influence their emotion to purchase (Cho and Fiorito, 2009). Emotions can be triggered by the content of digital signage, such as advertisements and editorial material, which can influence impulse purchasing behaviour (Garaus et al., 2017). Consequently, emotions are fundamental to the technological innovations in online customisation (Lang et al., 2021).

Researchers have also found that both integral emotions and incidental emotions strongly impact the customer’s judgments and decision making (Lerner et al., 2015). Both emotions and purchase behaviour can be impacted by congruent multisensory atmospheric cues in store, and the right emotions can enhance the consumer’s desire to purchase (Spence et al., 2014; Helmefalk and Hultén, 2017). Given the above, further research is undoubtedly still needed into the question of how multisensory perception and consumers’ emotions influence their desire to purchase. Researchers have already demonstrated that positive emotions are an important factor in online shopping for clothing (Lerner et al., 2015; Guo et al., 2020; Li et al., 2020).

The present research was designed to explore how consumer perceptions and emotion influence purchase intent and the way in which emotions mediate the relationship between perception and purchase intent. Specifically, the dimensions of arousal and perceived dominance can potentially be used to help explain the way in which emotions mediate purchase intent in the context of clothing e-customisation. Furthermore, we review the research linking multisensory perception and emotion. By integrating these fields of research, we demonstrate how multisensory perception and emotions play a key role in consumer intentions during clothing e-customisation. To assist e-retailers and brand managers in designing marketing strategies, the present study explores how multisensory perception influences positive emotions and purchase behaviour. The theoretical contributions of this research are twofold. First, the paper demonstrates how insights from multisensory perception and positive emotions can extend to purchase intent in e-customisation. More specifically, the paper presents an integrated conceptual research framework along with three main dimensions and provides a full understanding of emotions as a mediator of purchase intent. This can further enrich the existing research literature in e-customisation, while potentially also helping brand managers to explore consumers’ perception in the context of online shopping. Second, this study offers concrete suggestions for future research organised around perception, emotions, and purchase intent that might bias consumer behaviour. Both contributions pave the way for the development of theoretical work concerning how perception and emotion shape consumers’ responses to a wide range of online shopping, which should be considered by both public and private institutions. This research paper was designed to analyse and discuss the relationship between multisensory perception, emotions, and purchase behaviour in terms of e-customisation. In particular, we wanted to investigate how visual perception, haptic imagery, and perceived auditory stimulation impact consumers’ emotions and purchase intent; and how designers and how e-retailers can optimize the multisensory stimuli they provide to consumers in order to enhance the latter’s emotions and purchase behaviour in the e-customisation system from both the cognitive and behavioural perspectives.

## 2. Literature review and hypotheses

### 2.1. E-customisation

The concept behind e-customisation is to provide effective products and services to customers so as to meet their needs and provide them with a unique value (see Gilmore and Pine, 1997; Yan et al., 2020, for reviews). It is a strategy by which to handle the production, deliver an affordable product, and potentially increase the retailers’ profits at the same time (Ananto et al., 2022). Some companies have adopted product customisation, such as NIKE, Vans Custom and Dell. E-customisation provides information concerning details of the fabric, colours, styles, and patterns to consumers, so they can create personalized clothing (Li and Chen, 2018). Style (e.g., selecting colours, styles), customised fit, and function are the main categories in customisation (Lang et al., 2021). The 3D web virtual display technology of clothing also enables consumers to obtain their own personalized products. The latter can watch it online interactively in real-time (Zhu et al., 2018).

Nowadays, e-customisation has been adopted by fashion companies by involving consumers in the clothing design process *via* selecting colours, fabrics, decorations, and silhouettes (Kamali and Loker, 2002), so as to reduce the cost of production and increase the likelihood of consumer satisfaction (Jain and Sundström, 2021; Noor et al., 2022). In a case study of Vans Customs (a brand of an American skateboarding shoe), Lang et al. (2021) demonstrated that consumers were pleased and felt excited to make their own unique footwear in the context of online customisation. It is important to convey fashion product presentation (e.g., view product image, view colour image) and clothing description to consumers within the online shopping context as there are limited relevant physical and sensory experiences available to them in the online context (Jang and Burns, 2004). For retailers and brand managers, the interface of a product recommendation system should be developed based on a consideration of consumers’ experiences and interaction while shopping online (Ku et al., 2014; Jost and Süsser, 2020). One Nestle brand created personalized boxes of chocolates for consumers, and Laurent Freixe (the head of Nestle’s Zone Europe) found that people need to be involved in customisation and personalization for having their unique products (York, 2011). Indeed, consumers buy clothes online based on the stimuli presented within the digital system (Hu et al., 2016), and their subjective perceptions have been considered as an indicator of satisfaction in the customisation process (Wang et al., 2022). So, for example, adding product sound to the virtual try-on of noisy clothing in a virtual mirror has been shown to extend the customer’s interaction and possibly also to increase their willingness to pay (Ho et al., 2013). The findings from the relevant literature are presented in Table 1.

**Table 1:** Summary of the contributions of product e-customisation in marketing.

Researchers	Methodology	Context	Key findings
Senanayake and Little (2010)	Survey analysis, interviews, and case study	24 companies surveyed; *N* = 438	- Five critical points of customisation defined, including design, fabrication, fit, feature, and postproduction
Tangchaiburana and Techametheekul (2017)	Pearson correlation coefficient and stepwise multiple regression analysis	438 e-commerce customers; mass customisation	- Customisation significantly affected customers’ needs to design clothing types - Customisation, context and commerce had a significant effect on customers’ needs to design clothing parts
Jiang et al. (2014)	Multiple-group structural equation modelling analysis	*N* = 768 (i.e., 192 for each of the four cells)	- Perceived information sufficiency positively impacts knowledge value and process value in e-customisation - Perceived e-customisation value is influenced by the dynamic of the information process
Lee (2016)	Online survey, confirmatory factor analysis, metric invariance test	*N* = 327	- Perceived competence and autonomy increased positive emotions (pleasure and arousal) - Pleasure contributes to a positive attitude towards the customised products - Consumer knowledge partially moderates the relationship between cognitive needs fulfilment and emotions
Lee and Park (2009)	Empirical study, factor analysis, and path analysis	North American Internet shoppers (*N* = 204)	- The relationship between the history of purchases and service personalisation attitudes is partially supported - Positive attitudes enhance purchase intention in personalised online services - There are positive effects of subjective norms on positive attitudes - The relationship between subjective norms and purchase intentions is supported
Pallant et al. (2020)	Latent cluster analysis and factor analysis	Study 1: *N* = 489; Study 2: *N* = 394	- Following customisation preference and consumption patterns, four segments are identified, such as non-customisers, new customers, active customers and lapsing customers
Yoo and Park (2016)	Web-based survey, exploratory factor analysis, & metric invariance tests	*N* = 303	- In mass customisation, satisfaction is influenced by hedonic value, utilitarian value, creative achievement value and social value - Consumer’s past loyalty and need for uniqueness influence consumer value and satisfaction differently
Park et al. (2013)	Structural equation modelling	*N* = 321	- The need for uniqueness and status aspirations exert a significant impact over attitudes towards e-customised products - Perceived risk of online shopping has a mediating effect on purchasing e-customised products

### 2.2. Multisensory perception

Multisensory stimuli (explicit sensory perception) enrich consumers’ experiences by stimulating one or more of their senses (i.e., visual, acoustic, haptic, olfactory and gustatory). Sensory perception should be considered by retailers in marketing strategy, who wish to gain a good understanding of consumer behaviour (Haase et al., 2018). In the context of a sportswear store visual, olfactory and tactile stimuli have been shown to exert a significant effect on female consumers’ purchase behaviour and attitudinal loyalty than males (Kartop and Ekizoglu, 2022). In terms of a stimulus–response framework, it has been shown that there is a significant relationship between consumers’ experiences and the sensory aspects of the online environment (Yoganathan et al., 2019). Supported by the Internet of Things (IoT) devices, the auditory, visual, and olfactory senses impact guest’s emotions and affective experiences while staying in upscale hotels (Pelet et al., 2021). Especially in a store environment, multisensory (e.g., auditory, olfactory) cues are often more effective at creating the desired store atmosphere than when only visual stimuli are used in the context of retail (Helmefalk and Hultén, 2017). In the context of online fashion shopping, haptic information can be conveyed *via* verbal descriptions and images that may encourage consumers to imagine feeling (or the feeling of) the product (Silva et al., 2021). Sensory integration affects consumers’ judgments, which should be considered by those wanting to try and understand consumer behaviour.

Multisensory experience and cues combine to influence purchase behaviour and emotions (Helmefalk and Berndt, 2018; Li et al., 2020). After exploring how information is presented in the environment by using congruent cues *via* technology, previous researchers have reported that consumers’ liking and behaviour are influenced by visual, haptic, and auditory stimuli (Beudaert et al., 2017; Liu et al., 2018). Researchers have also found that multisensory congruence and perception exerts a significant impact on consumers’ decision-making in the case of clothing customisation (Senanayake and Little, 2010; Helmefalk and Hultén, 2017; Petit et al., 2019). Summary of the multisensory studies are presented in Table 2.

According to the emerging field of sensory marketing, sensory cues influence consumers’ cognition, affect, and behaviour (Spence et al., 2014). When they feel better and are satisfied, they tend to exhibit more favorable behaviour in the context of retailing. Meanwhile, prior studies have distinguished between emotion and purchase intent to buy clothing within an online shopping environment (Tangchaiburana and Techametheekul, 2017; Li et al., 2020). Sensory experiences enhance the value of online interactions when shopping for clothes (Kim and Forsythe, 2009). For instance, the same chocolate was rated as tasting sweeter while listening to positive rather than negative music, and people’s self-reported purchase intent was also higher (Reinoso-Carvalho et al., 2020a). By exploring how sensory information is presented in the immersive testing environment, researchers have previously reported that what consumer’s liking of cold brewed coffee, is both influenced by the sources of visual and auditory stimuli (Liu et al., 2018). More senses may provide additional information for consumer decision-making and increase purchase behaviour (Morrin and Chebat, 2005; Krishna, 2012).

Previous investigations of multisensory experiences in the context of online shopping have tended to focus largely on perceptions of stimuli, such as colour and other aspects of visual appearance. But some findings have revealed that sensory overload may interfere with the consumers’ evaluation and behaviour (Malhotra, 1984; Doucé and Adams, 2020). Considering the current situation in product e-customisation, sensory information may, or may not, make products more attractive to consumers. Therefore, this study attempts to explore the relationships between multisensory perception, emotions and purchase behaviour in the context of e-customisation.

**Table 2:** Summary of studies that have investigated the effect of multisensory perception on texture.

Study	Stimulus	Sensory modality	Topics of experiments	Findings
Guest and Spence (2003)	Fabrics	Visual & tactile	Experiment 1: Visual, tactile and bimodal (visual and touch) on roughness Experiment 2: A visual or tactile stimulus with another bimodal stimulus intervening Experiment 3: Both single (visual or touch) and bimodal were adopted to assess rough or smooth	Congruent bimodal (visual and touch) roughness information did not improve roughness discrimination Vision, touch, and bimodal perception provide information about roughness Both visual and touch inputs contribute to decision making
Kim et al. (2019)	Texture	Auditory, tactile, & visual	Five different stimuli used to evaluate each modality. Surface stickiness tested using vision, touch, and audition	Visual and tactile perception are similar, but auditory is different as it has a better discrimination of stickiness Concerning texture information processing, audition is different from the other senses (visual or touch) Best to assess stickiness using audition, as sound is distinctive from the other modalities (visual and touch)
Krishna et al. (2010)	Texture	Olfactory & tactile	Study 1: Focused on smell and touch across genders Study 2: Focused on smell and touch at different temperatures	Olfaction impacts touch Multisensory semantic congruence enhances product evaluation and haptic perception Smell and touch congruence influences behaviour and experiences
Ishikawa et al. (2015)	Photo images of fabric, fabric samples	Visual & tactile	Experiment 1: Visual perception Experiment 2: Both visual and tactile perception	The photography method can be used to assess the thickness of fabric samples Visual perception and visual-tactile perception are very similar to fabric texture recognition

#### 2.2.1. Visual perception

Vision is the dominant sense in humans (Gallace et al., 2012). In fact, it has been estimated that 80% of stimuli are received visually in online environments (Hutmacher, 2019). The attractive design of websites consists of easily-understandable web browsing and the attractive use of colour, font/typeface size, and photographs on the website (Cachero-Martínez and Vázquez-Casielles, 2021). The effect of Facebook advertising positively associated with purchase intention (Jermsittiparsert, 2019). Some studies indicate that the quality of visual display refers to the website quality, colours, the aesthetics of the product display, and visual appeal (Liu et al., 2013; Bressolles et al., 2014; Kimiagari and Malafe, 2021). When shopping for clothes online, the colour and shape of the displayed items can capture people’s attention (Mo et al., 2021). Retailers provide e-customised information for clothing, such as information about the textiles, colours, sizes, patterns and sewing technology involved.

According to Moshagen and Thielsch (2010), the aesthetic quality of websites can be measured by the four interrelated facets of simplicity, diversity, colourfulness, and craftsmanship. So, for example, consumers have been shown to pay more visual attention to light-rather than dark-coloured products when listening to high-frequency sounds (Hagtvedt and Brasel, 2016; Yang et al., 2022). The given colour value (darkness or lightness) also influences consumers’ visual perception on the products’ weight and density (Hagtvedt, 2014). In particular, both colour matching and the products’ exterior design can influence consumers’ customisation experiences (Li and Liu, 2020). The visual aesthetics (formality, appeal) of websites directly impacts customer satisfaction and purchase behaviour (e.g., Wang et al., 2011). Those factors are essential when it comes to attracting the consumers’ attention. The usefulness and informativeness of the website influences the consumers’ attitude toward it and their purchase intent (Hausman and Siekpe, 2009). Supported by the stimulus-organism-response model, Li et al. (2022) suggest that consumers’ impulsive purchase intentions are positively affected by their visual perception on apparel attributes and fashion information in e-customisation. Colour provokes consumers’ physiological and emotional responses, as warm colours are associated with cheerful moods (Roschk et al., 2017). The effects of the visual, navigational, and informational characteristics of website design on consumers’ perceptions when shopping online may help to arouse and maintain the attraction of websites (Hasan, 2016). This current study will investigate whether visual perception influences purchase intent, arousal, and dominance. Since displayed clothing items, fashion information, and perceived visual aesthetic of the website are likely to affect consumers purchase behaviour (Wang et al., 2011; Mo et al., 2021; Li et al., 2022), the hypotheses are presented as follows,

*H1a*. Visual perception has a significant effect on purchase intent in e-customisation.

*H2a*. Visual perception has a significant effect on arousal when the customer is engaged in e-customisation.

*H3a*. Visual perception has a significant effect on dominance when the customer is engaged in e-customisation.

#### 2.2.2. Perceived auditory stimulation

Music is considered to be an important component of the shopping environment and of the consumer’s experience (Cuny et al., 2015), and the loudness and tempo of the background music has been shown to increase the consumers’ engagement in the simulated immersive 3D environment (Papagiannidis et al., 2017). Meanwhile, in the context of the shopping mall, background music enhances the consumers’ pleasure and dominance emotions and positive evaluations of the environment (Yi and Kang, 2019). In the real-world, high-tempo music may bemore likely to encourage the consumer to purchase products than any change to the ambient lighting (Hagtvedt and Brasel, 2016). The selection of products by the consumer can sometimes be strongly influenced by auditory stimuli (stereotypical French and German music) in a web shopping environment (Damen et al., 2021), here matching the findings reported previously showing that auditory sensory cues affect consumers’ emotion and purchase decisions in the store atmosphere (Yalch and Spangenberg, 2000; Helmefalk and Hultén, 2017). Auditory cues can be used to bias a consumer’s visual product search, attention, selections and decision-making (Knoeferle et al., 2016).

Music congruency in a service setting enhances pleasure and may increase consumers’ evaluation of service quality, in turn directly affecting the latter’s repurchase intent (Demoulin, 2011). Crossmodally congruent background music leads experiential browsers to have more positive reactions than incongruent ones (Doucé et al., 2022). In the e-commerce environment, interactive music that provides individuals with the opportunity to modify the melody and/or tempo, may create positive experiences for consumers and thus increase their engagement and behavioural intention (Hwang and Oh, 2020). Similarly, soft music (i.e., music having a slow tempo, consonant harmony, low volume, and smooth-flowing rhythms) can influence the consumers’ haptic perceptions of product softness and even their willingness to pay (Imschloss and Kuehnl, 2017, 2019). Furthermore, perceived voice control with voice-activated devices (e.g., Microsoft HoloLens) positively influences consumers’ intent to pay, and congruent auditory feedback moderates decision-making in online shopping (Heller et al., 2019).

Emotional states, in particular, are influenced by tempo and tone of auditory stimulation in online shopping. The tempo of the music and any background sounds/music may affect arousal and pleasure, which then affects online shopping behaviour (Ding and Lin, 2012). Higher arousal can be induced by fast-tempo music as compared to slow-tempo music (Zhu and Meyers-Levy, 2005; Day et al., 2009). For example, through testing the control group (no sound stimulation) and the experimental group (with audio stimulation), Hultén (2015) found that the introduction of auditory cues incorporating human voice positively influence shopping behaviour and emotions amongst both parents and their children in the setting of a retail grocery. After surveying 70 United Kingdom web sites, Fiore and Kelly (2007) found that sound is used to remind the customer about fashion product features, communicate with them and affect their mood in the online context. Background music has been used to create a pleasant environment and high-frequency sounds affect visual attention towards light-coloured objects, which may influence consumers’ shopping behaviour and mood (Gorn et al., 1991; Hagtvedt and Brasel, 2016). Auditory stimuli and background music also affect consumers’ perceptions and decision-making through the modulation of emotion (Zhu and Meyers-Levy, 2005). Therefore, it is possible that perceived auditory stimulation may directly influence consumers’ emotions and purchase intent when shopping online, because the tempo, pitch, and tonality of the music are influential factors to emotion and decision-making (Zhu and Meyers-Levy, 2005; Hagtvedt and Brasel, 2016; Imschloss and Kuehnl, 2017). The hypotheses are presented as follows:

*H1b*. Auditory stimulation has a significant effect on purchase intent in e-customisation.

*H2b*. Auditory stimulation has a significant effect on arousal when the customer is engaged in e-customisation.

*H3b*. Auditory stimulation has a significant effect on dominance when the customer is engaged in e-customisation.

#### 2.2.3. Haptic imagery

Stimulating the sense of touch (even indirectly, i.e., *via* tactile/haptic mental imagery) is important in online shopping experiences (Brasel and Gips, 2014; see also Lacey et al., 2012). In the virtual world, engaging the customer’s sense of touch/haptics *via* the interface (e.g., screen touch, mouse touch) has been shown to increase the interactivity of images as well as the consumers’ feelings of ownship (Vries et al., 2018). It has been suggested that women may need more tactile information (verbal and visual information) than men in online product evaluation and purchase decision-making (Citrin et al., 2003). Activating haptic mental imagery can be used to help increase their memory for pictures (Paivio, 1975), as well as allow them to get a sense of how textures/surfaces would feel (Witmer et al., 2005). When making judgments and selections, consumers rely more on imagery than textual content (Kim and Kim, 2022). The personal assessment can also be made *via* haptic imagery (Petkova et al., 2011). In the context of online fashion shopping, haptic imagery may involve imagining the texture of products instead of actually touching it, which can create a feeling that consumers are familiar with (Choi and Taylor, 2014; Rodrigues et al., 2017; Bhatia et al., 2022). The experience of touching has been shown to influence the consumers’ selection, emotions and evaluation of products (Krishna et al., 2010; Streicher and Estes, 2016).

Driven by the development of technology, screen touch is becoming increasingly common in online shopping (Mulcahy and Riedel, 2020; Canio and Fuentes-Blasco, 2021). The website has an indirect touch interface *via* a mouse, the mobile application provides direct touch (e.g., touch-screen, touch-pad) to consumers (Kühn et al., 2020). Further, touching the screens on an iPad has been shown to exert a positive effect on intentions and behaviours (Brasel and Gips, 2015). For instance, when touching clothes online, sensory information (e.g., the image of texture) may be transferred by means of automatically-triggered crossmodal haptic imagery (Spence and Deroy, 2013; Huang and Liao, 2017). Further, haptic perception functions as a mediator in human-machine interfaces and influences consumers’ purchase intent (Gallace and Spence, 2014). Compared to the non-haptic touch condition (watching and filling-in in the questions), haptic touch is becoming increasingly important within the digital shopping environment, which enable consumers to interact with products by, for example, shaking their mobile phone and swiping on it (Mulcahy and Riedel, 2020). Pictorial and verbal information can generate haptic imagery, and influence purchase intent (Silva et al., 2021).

In physical retail settings, engaging the customers’ sense of touch/haptics has been shown to increase purchase intent, as it provides tactile stimulation and therefore to give emotional experiences to individuals (Spence and Gallace, 2011; Gallace and Spence, 2014; Hutmacher, 2019). Even though consumers cannot practically touch realistic textures in an online fast fashion store, the dynamic mix-and-match can arouse more mental images of product than static pictures or verbal descriptions (Overmars and Poels, 2015; Rodrigues et al., 2017). When having multisensory experiences using augmented-reality (AR) interactive technology, Huang and Liao (2017) found that haptic imagery helps to create the sense of ownership over a realistic product, which positively influenced self-exploratory engagement in multisensory experiences. In the online shopping environment, haptic images are recommended to consumers who can get information about the fabric and other attributes of the clothing, which influences their purchase intent (Ha et al., 2007; Rodrigues et al., 2017). Therefore, haptic imagery can influence consumers’ emotion and purchase intent *via* pictorial and verbal information in online retailing (Huang and Liao, 2017; Silva et al., 2021). The present study was also designed to investigate haptic imagery in order to examine its effects on emotions and purchase intent. The hypotheses are presented as follows,

*H1c*. Haptic mental imagery has a significant effect on purchase intent in e-customisation.

*H2c*. Haptic mental imagery has a significant effect on arousal when the customer is engaged in e-customisation.

*H3c*. Haptic imagery has a significant effect on dominance when the customer is engaged in e-customisation.

### 2.3. Emotions

Emotions are defined in terms of an individual’s affective responses to situations that are influenced by communication, technical innovation, and their experiences (Gezhi and Xiang, 2022; Mimoun et al., 2022). Emotions refer to mental state related to cognitive events and/or thoughts and can influence an individual’s behavioural intentions (Bagozzi et al., 1999, p. 184; Yan et al., 2018). The pleasure-arousal-dominance (PAD) model has been used to capture an individual’s emotional state. Pleasure relates to a person’s mood change from unhappiness to happiness (Russell and Mehrabian, 1977), while arousal refers to the individuals’ level of excitement or the extent that they feel stimulated. Dominance refers to the degree of an individual feels free or in control.

The perceived visual, haptic and auditory stimuli impact pleasure valence and arousal (Huang et al., 2017). In terms of online shopping, pleasure, arousal and dominance mediate the relationship between website atmosphere and purchase intent (Hsieh et al., 2014). Supported by the PAD model, Graa and Dani-el Kebir (2012) found that consumers’ pleasure had a mediating effect on their purchase behaviour. The shopping experience includes the interactions between consumers and products, interfaces and brands at the emotional and sensory levels (Lemon and Verhoef, 2016). Lyubomirsky (2007) and Seligman (2002) concluded that there are three categories of positive emotions: the past (satisfaction), the present (pleasure and gratification), and the future (optimism). Pleasure is an emotional response that involves a feeling of joy or satisfaction (Ward and Barnes, 2001; Eroglu et al., 2003).

Similarly, researchers found that there are two independent dimensions of positive emotions, namely valence and arousal (Posner et al., 2005). Arousal has been proposed as a measure for emotions (Mehrabian and Russell, 1974). Indeed, perceived pleasure can be determined by arousal, valence, and dominance (Zhang et al., 2017). Within the context of e-customisation, multisensory perception has an effect on positive emotions and may influence perceived pleasure (Helmefalk and Hultén, 2017). In retail settings and store atmospheres, different multisensory stimuli impact emotions and purchase intent (Spence et al., 2014; Helmefalk and Hultén, 2017). After using eye-tracking, a survey and interviews, Cyr et al. (2010) found that the colour of the interface or apparel on screens affect an individual’s emotions, their satisfaction, and their trust in a website. The literature shows that pleasure influences behaviour *via* experiences and activities, such as experiencing art and music (Bolonkin, 2012). Obviously, arousal and dominance may influence purchase intent in e-customisation (Hsieh et al., 2014). Thus, the hypotheses are presented as follows:

*H1d*–*H1e*. Arousal (*H1d*) and dominance (*H1e*) significantly influence purchase intent when the customer is engaged in e-customisation.

*H4a*–*H4c*. Arousal mediates the effect of visual perception (*H4a*), auditory stimulation (*H4b*), and haptic mental imagery (*H4c*) on purchase intent when the customer is engaged in e-customisation.

*H5a*–*H5c*. Dominance mediates the effect of visual perception (*H5a*), perceived auditory stimulation (*H5b*), and haptic mental imagery (*H5c*) on purchase intent when the customer is engaged in e-customisation.

## 3. Methodology

**Figure 1:**
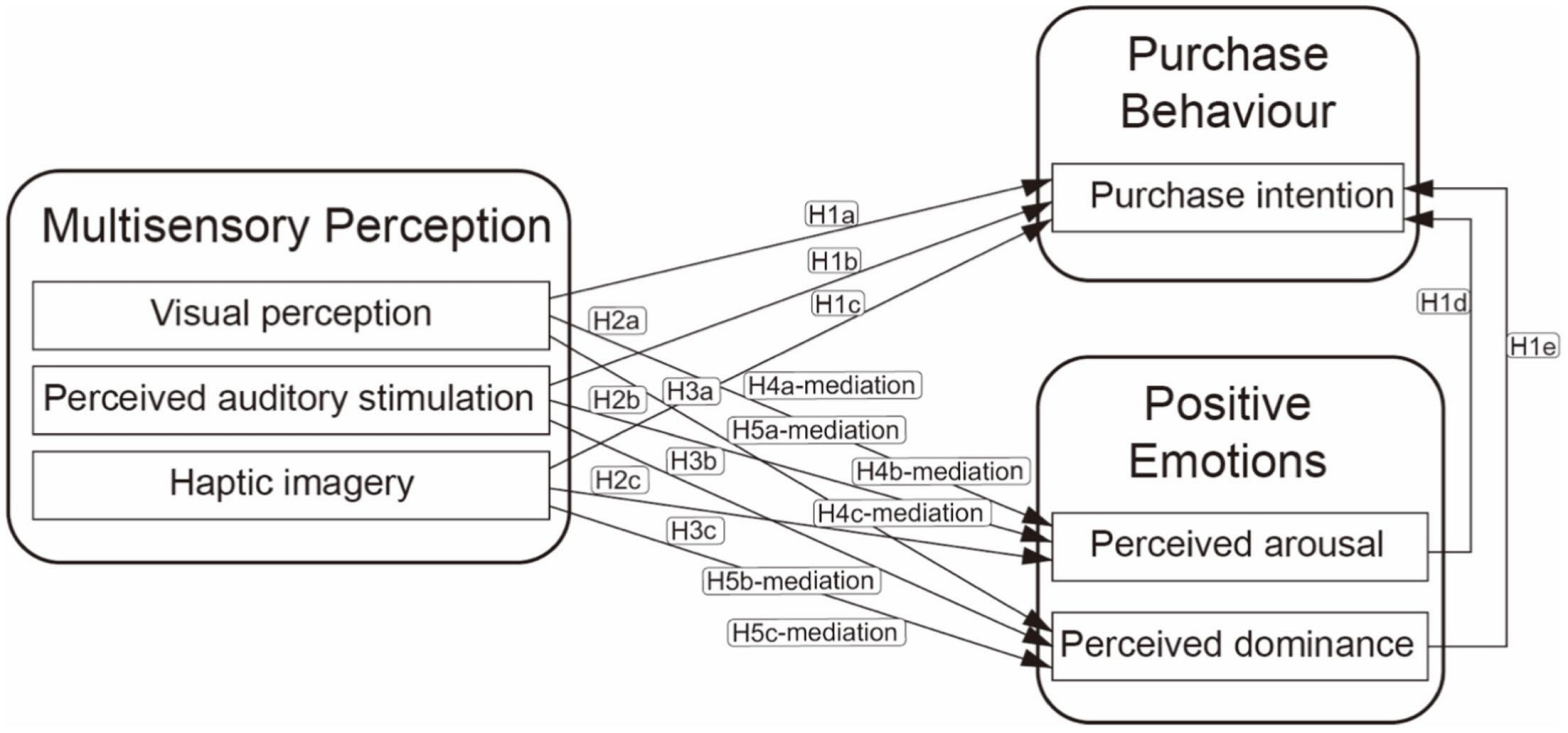
Multisensory perception, positive emotions, and purchase behaviour in an e-customisation (MPP-E) conceptual framework.

The current research was designed to investigate the importance of multisensory perception, emotions, and purchase intent in the context of fashion online shopping. The conceptual framework outlines the relationship between multisensory perception (e.g., of visual, haptic, and auditory stimuli), emotions (perceived arousal, perceived dominance), and purchase intent (see Figure 1). The study demonstrates that the fashion customisation process should enhance the customers’ emotions, as this may have a knock-on effect on decision-making. This study also demonstrates that consumers’ perception and emotion may influence their purchase intent.

## 4. Experiment

The study reported here had two main objectives. First, it was designed to investigate whether multisensory perception in fashion e-customisation technology would increase participants’ emotions. Second, the effects of visual perception, haptic mental imagery, and perceived auditory stimulation on emotions (arousal, dominance) were explored. The auditory stimuli were presented during the experiment with the same tempo, pitch, and tonality. The objective was to meet the requirements of e-customisation technology for online fashion customisation. The participants were selected randomly, with each participant being asked to use e-customisation (e.g., Cotte Yolan, Spreadshirt, YUNYIDINGZHI, Uniqlo) in order to customise a fashion product prior to taking part in the study.

### 4.1. Method

#### 4.1.1. Sample and design

This experiment followed the ethical principles and laboratory safety regulations of Shanghai University of Engineering Science. To keep the accuracy of the questionnaires, two bilingual speakers (Chinese and English) translated the questions, and one language expert check the translated items. All of them were informed that the questionnaires were about multisensory perception and purchase intent in clothing e-customisation. The participants were chosen at random and all took part voluntarily. They had to complete the study in a quiet and relaxed environment. Before completing the questionnaire, they were instructed to customise the product for themselves or for someone else online *via* selecting detailed section of styles, colours, patterns, and decorations. There was no time limit associated with filling out the questionnaire. The sample size was 398 (see Table 3 for the descriptive statistics for the collected sample).

**Table 3:** Descriptive statistics.

Variable and categories	*N*	Percent
**Gender**		
Male	220	55.3
Female	178	44.7
**Education**		
High school	12	3.0
College	60	15.1
Bachelor’s degree	195	49.0
Master’s degree	128	32.2
Doctoral degree	3	0.7
**Age (years)**		
Less than 18	15	3.8
18–23	225	56.5
24–29	137	34.5
30–35	15	3.8
36–41	3	0.7
42 and over	3	0.7
**Monthly income (Chinese yuan)**		
Less than 2000	214	53.8
2001–4,000	74	18.6
4,001–6,000	36	9.0
6,001–8,000	23	5.8
8,001–10,000	24	6.0
10,001 and above	27	6.8
**Online shopping experience**		
Less than 2 years	26	6.5
3–4 years	103	25.9
5–6 years	176	44.2
7–8 years	61	15.3
9–10 years	19	4.8
More than 10 years	13	3.3
**Shopping frequency**		
Never	0	0.0
1–2 times per week	131	32.9
3–4 times per week	209	52.5
Once per day	20	5.1
Several times per day	38	9.5
**Monthly amount spent on clothes (Chinese yuan)**		
Less than 200	92	23.1
201–400	116	29.2
401–600	98	24.6
601–800	42	10.6
801–1,000	32	8.0
1,001 and above	18	4.5

#### 4.1.2. Measures

Measurement scales were adapted and revised from the literature for use in the study. The three visual perception scales (VIPE) were adapted from Webster and Macleod (1988), Vieira (2010), and Jang et al. (2018). The four haptic imagery scales (HAIM) were obtained from Longo et al. (2008) and Witmer et al. (2005). The three auditory stimulation scales (PADS) were derived from the studies of Demoulin (2011) and Dube and Morin (2001). The six scales used to measure arousal (PARS) and dominance (PDMN) were based on Yang et al.’s (2020) study. The three scales used to measure purchase intent (PIN) were obtained from Pavlou (2003). The scale measures are presented in Appendix A.

#### 4.1.3. Procedure

The participants were recruited by email and WeChat, and the study was described as a ‘sensory marketing experiment’. The participants first had to choose a website (e.g., Cotte Yolan, Spreadshirt, YUNYIDINGZHI, Uniqlo) and design a fashion product using the e-customisation system. Meanwhile, one of the auditory stimuli (e.g., Canon in D Major, Por Una Cabeza, Summer) was chosen randomly and played to the participant while they interacted with the system. The auditory stimuli were chosen from Net Ease Cloud Music. They were played on a mobile phone with the participants selecting the volume that they preferred. The participants were then asked to fill out the questionnaires on e-customisation without time limit. The items were measured on a five-point Likert scales, with 1 = ‘strongly disagree’ and 5 = ‘strongly agree’. Finally, demographic information was collected.

### 4.2. Data analysis

#### 4.2.1. Reliability and validity

The exploratory factor analysis was conducted to test the reliability of the data. The threshold for the factor loadings was above 0.5 (Gefen et al., 2000), and the threshold for the composite reliability was 0.7 (Nunnally, 1978a). The internal reliability of the scales was assessed using Cronbach’s alpha, and the threshold should be greater than 0.7 (Nunnally, 1978b; Hair et al., 2019). The average variance extracted value of the constructs was determined to exceed 0.5 (Fornell and Larcker, 1981).

In this study, the visual perception scales (3 items, Cronbach’s alpha = 0.754), auditory stimulation (3 items, Cronbach’s alpha = 0.816), haptic mental imagery (4 items, Cronbach’s alpha = 0.789), arousal (3 items, Cronbach’s alpha = 0.761), dominance (3 items, Cronbach’s alpha = 0.701), and purchase intent (3 items, Cronbach’s alpha = 0.776) were tested in SPSS 23.0. As recommended by Hair et al. (1998), the reliability was good, as was the factor loadings (FL > 0.7), the average variance extracted (AVE > 0.5), the Kaiser-Meyer-Olkin measure of sampling adequacy (KMO = 0.845) and the Bartlett’s test (significant at *p* < 0.05, *df* = 171, approximate Chi-square = 2357.156). A factor analysis confirmed that the scales were valid (see Table 4).

**Table 4:** Exploratory factor analysis.

Construct	Code	Cronbach’s alpha	Factor loading	CR	AVE
Visual perception	VIPE1	0.754	0.701	0.798	0.570
	VIPE2		0.728		
	VIPE3		0.830		
Haptic imagery	HAIM1	0.789	0.701	0.825	0.542
	HAIM2		0.797		
	HAIM3		0.729		
	HAIM4		0.715		
Perceived auditory stimulation	PADS1	0.816	0.816	0.859	0.669
	PADS2		0.823		
	PADS3		0.815		
Perceived arousal	PARS1	0.761	0.716	0.773	0.532
	PARS2		0.734		
	PARS3		0.737		
Perceived dominance	PDMN1	0.701	0.701	0.755	0.506
	PDMN2		0.702		
	PDMN3		0.731		
Purchase intent	PIN1	0.776	0.794	0.817	0.599
	PIN2		0.782		
	PIN3		0.744		

VIPE, visual perception; HAIM, haptic imagery; PADS, perceived auditory stimulation; PARS, perceived arousal; PDMN, perceived dominance; PIN, purchase intent.

The correlation coefficients of the variables were above 0 and less than the square root of AVE, indicating that each of the latent variables was correlated with the others (see Table 5). The discriminant validity of the data was good.

**Table 5:** Correlation analysis and discriminant validity test among variables.

	VIPE	PADS	HAIM	PARS	PDMN	PIN	VIF
VIPE	0.570*						1.398
HAIM	0.452**	0.542*					1.404
PADS	0.398**	0.325**	0.669*				1.250
PARS	0.276**	0.257**	0.311**	0.532*			1.246
PDMN	0.163**	0.126*	0.296**	0.337**	0.506*		1.182
PIN	0.397**	0.350**	0.424**	0.346**	0.249**	0.599*	-

**Correlation is significant at the 0.01 level (2-tailed); *Correlation is significant at the 0.05 level (2-tailed). Maximum variance method; VIF, variance inflation factor.

#### 4.2.2. Regression analysis

##### 4.2.2.1. The influence of multisensory stimulation on purchase intent

A regression analysis revealed that the *F*-value of the sample was 75.655, and the significance of visual perception (*p* < 0.001), auditory stimulation (*p* < 0.001) and haptic imagery (*p* < 0.001) to purchase intent was valid. This result indicates that the linear relationship between the independent and dependent variables of the regression equation was significant. The goodness of fit of the regression equation was good (*R*^2^ = 0.558), thus suggesting that the independent variable could explain the dependent variable to a high degree.

The results revealed that visual perception (*β* = 0.341, *t* = 7.856, *p* < 0.001), haptic imagery (*β* = 0.276, *t* = 6.356, *p* < 0.001), auditory stimulation (*β* = 0.256, *t* = 5.904, *p* < 0.001), arousal (*β* = 0.187, *t* = 4.090, *p* < 0.001), and dominance (*β* = 0.243, *t* = 4.974, *p* < 0.001) significantly influenced purchase intent. Therefore, *H1*a, *H1*b, *H1*c, *H1*d, and *H1*e are supported (see Table 6).

**Table 6:** Regression results for multisensory perception and purchase intent.

Hypothesis	Direct effects	Beta	*t*	value of p	Decision
H1a	VIPE→ PIN	0.341	7.856	***	Supported
H1b	PADS→ PIN	0.256	5.904	***	Supported
H1c	HAIM→ PIN	0.276	6.356	***	Supported
H1d	PARS→ PIN	0.349	7.409	***	Supported
H1e	PDMN→PIN	0.243	4.974	***	Supported
	*F*	75.655 (*p* = 0.000)			
	*R* ^2^	0.558			

***p < 0.001.

##### 4.2.2.2. The influence of multisensory stimulation on arousal and dominance

Visual perception directly affected arousal (*β* = 0.257, *t* = 5.481, *p* < 0.001), while haptic imagery directly affected arousal (*β* = 0.187, *t* = 3.998, *p* < 0.001). Auditory stimulation also directly affected arousal (*β* = 0.187, *t* = 3.999, *p* < 0.001). Visual perception directly affected dominance (*β* = 0.270, *t* = 5.612, *p* < 0.001). Haptic imagery directly affected dominance (*β* = 0.095, *t* = 1.979, *p* < 0.05). There was no significant relation between auditory stimulation and dominance (*β* = 0.070, *t* = 1.457, *p* = 0.146). Therefore, *H2*a, *H2*b, *H2*c, *H4*a and *H4*b are supported, whereas *H4*c was not proven (see Table 7).

**Table 7:** Test of direct effects.

Hypothesis	Direct effects	Beta	*t*	value of *p*	Decision
H2a	VIPE→PARS	0.257	5.481	***	Supported
H2b	PADS→PARS	0.187	3.999	***	Supported
H2c	HAIM→PARS	0.187	3.998	***	Supported
H3a	VIPE→PDMN	0.270	5.612	***	Supported
H3b	PADS→PDMN	0.070	1.457	0.146	Unsupported
H3c	HAIM→PDMN	0.095	1.979	0.049*	Supported

***p < 0.001, *p < 0.05.

#### 4.2.3. Mediation analysis

Process mediated effect test (Model 4, mediated model) in SPSS was adopted for the analysis. Relative proximity (RP) provides a degree of significance for confidence intervals (Götz et al., 2021). The valid confidence intervals do not overlap with zero (Hayes, 2013; Charlton et al., 2021). The results revealed that perceived arousal had a mediating effect between visual perception and purchase intent (*t* = 4.962, *p* < 0.001). Arousal had a mediating effect between auditory stimulation and purchase intent (*t* = 5.221, *p* < 0.001). Arousal also mediated between haptic imagery and purchase intent (*t* = 4.531, *p* < 0.001). Dominance mediated between haptic imagery and purchase intent (*t* = 2.226, *p* < 0.05). Dominance did not mediate visual perception and purchase intent (*t* = 1.148, *p* = ns). Dominance did not mediate perceived auditory stimulation and purchase intent (*t* = 0.656, *p* = ns). Thus, *H3*a, *H3*b, *H3*c and *H5*c are supported, while *H5*a and *H5*b are not (Table 8).

**Table 8:** Assessing mediation effects.

Hypothesis	Construct		*T*-value	Effect	BootSE	BootLLCI	BootULCI	RP	Decision
H4a	VIPE→PARS→PIN	Direct	4.222***	0.226	0.054	0.121	0.331	0.574	Full mediation
		Mediation	4.962***	0.050	0.018	0.020	0.090	0.287	
		Total	5.144***	0.276	0.054	0.170	0.381	0.808	
H4b	PADS→PARS→PIN	Direct	3.615***	0.205	0.057	0.094	0.317	0.419	Full mediation
		Mediation	5.221***	0.051	0.018	0.023	0.095	0.312	
		Total	4.390***	0.256	0.058	0.142	0.371	0.617	
H4c	HAIM→PARS→PIN	Direct	4.845***	0.285	0.059	0.170	0.401	0.732	Full mediation
		Mediation	4.531***	0.056	0.017	0.028	0.097	0.410	
		Total	5.782***	0.341	0.059	0.225	0.457	0.970	
H5a	VIPE→PDMN→PIN	Direct	5.000***	0.265	0.053	0.161	0.370	0.772	Not mediation
		Mediation	1.148	0.011	0.010	−0.007	0.035	−0.157	
		Total	5.144***	0.276	0.054	0.170	0.381	0.808	
H5b	PADS→PDMN→PIN	Direct	4.365***	0.250	0.057	0.137	0.362	0.610	Not mediation
		Mediation	0.656	0.007	0.011	−0.011	0.032	−0.260	
		Total	4.390***	0.256	0.058	0.142	0.371	0.617	
H5c	HAIM→PDMN→PIN	Direct	5.155***	0.314	0.061	0.194	0.434	0.811	Full mediation
		Mediation	2.226*	0.027	0.014	0.004	0.060	0.077	
		Total	5.782***	0.341	0.059	0.225	0.457	0.970	

*p < 0.05; **p < 0.01; ***p < 0.001; ns: not significant.

## 5. Discussion

Nowadays, customers often purchase products to meet their personal requirements, and often they do not have either the opportunity or desire to purchase fashion products from bricks-and-mortar stores. Supported by internet technology and emerging customization systems, retailers are increasingly able to provide services for those consumers who choose to select and purchase their clothes online. Optimizing the multisensory stimulation of customers’ in e-customisation is vital for retailers. In the process of fashion customisation, customers are encouraged to select the unique details of their clothes, and get recommendations concerning the products. In this way, consumers’ purchase intent can be triggered by multisensory (e.g., visual and auditory) stimuli.

The relationship between multisensory perception and purchase intent has been studied by many researchers, but there is far less research linking visual perception, haptic imagery, and perceived auditory stimulation with purchase intent in fashion e-customisation, and none that relates specifically to the mediation effect of perceived arousal and perceived dominance. First, after testing the effects of visual perception on purchase intent, visual perception has a significant effect on purchase intent (*H1*a), perceived arousal (*H2*a), and perceived dominance (*H3*a). The three hypotheses were accepted. This is in line with previous research by Babin et al. (2003), Hekkert (2006), Jin (2009), Park et al. (2008), and Yim et al. (2017), who all found that virtual experience positively impacts consumers’ pleasure, online shopping behaviour, selection, and willingness to buy when purchasing products. For brand managers, the outcomes of this study are important as far as targeting consumers’ perceptions in e-customisation is concerned. Various functions and stimuli are shown to consumers that can influence their purchase intent. Some consumers may be attracted by more colour matching and aesthetics, while others rely on the product adjustment and perceived music. This suggests that consumers are willing to match colour and form online and that the visual aesthetics of a website and/or mobile application do indeed influence purchase intent.

Second, perceived auditory stimulation has a significant effect on purchase intent (*H1*b) and perceived arousal (*H2*b). The two hypotheses were accepted. This also supports the previous findings of Andersson et al. (2012), who demonstrated that auditory stimulation influences consumers’ emotions and purchase decisions. However, auditory stimulation does not have significant effects on dominance, as *H3*b was not supported, which suggests that music tempo, pitch and tonality were not directly related to dominance in the e-customisation environment, in line with Lazaris et al. (2022) previous findings. The latter’s findings proved that consumers’ affective responses (e.g., dominance) were not stimulated by omnichannel atmosphere (offline, online). This means that music tempo, pitch and tonality might not affect pleasure. It is possible that some consumers might not have audio tools or be able to listen to audio or they may find listening to it inconvenient. Thus, for online retailers, this research provides evidence that may be useful when designing online stimuli as it is important to consider the related sensory cues in the process of fashion e-customisation.

Third, haptic mental imagery has significant effects on purchase intent (*H1*c), perceived arousal (*H2*c) and perceived dominance (*H3*c). In line with Michel et al. (2017) previous findings, the three hypotheses that music would affect consumers’ emotions were accepted. So, for online retailers, this research provides useful evidence when designing online stimuli as it is important to consider the related sensory cues in the e-customisation process. For instance, brand managers should provide consumers with their preferred textile and personalized interfaces according to the view of consumers’ perception to arouse their pleasure, instead of merely focusing on the designer’s inspirations and/or creative ideas. Our findings are also in line with the previous findings of Rodrigues et al. (2017) and Huang and Liao (2017), who both reported that fabric and haptic information can be conveyed *via* images and information that can contribute to the recalling of consumers’ past experiences and also influence their emotions. Although visual perception and haptic imagery directly affect purchase intent, as hypothesized, colour matching and adjustments on the screen are also significantly related with emotions. For online retailers, this indicates that the aesthetic of online websites and clothing adjustment functions are more important than verbal description as far as increasing consumers’ pleasure during their e-customisation experiences is concerned.

Fourth, both arousal (*H1*d) and dominance (*H1*e) exert significant effects on purchase intent in the context of e-customisation. The two hypotheses were supported, which is in line with Hsieh et al’s. (2021) previous findings. They identified that arousal and dominance can affect users’ intentions in brand apps. This is demonstrated by the impact of arousal and dominance on the e-customisation process. Brand managers should therefore focus their efforts on increasing consumers’ pleasure and on the effect of multisensory cues in the fashion e-customisation environment. For example, the use of well-designed interface, auditory stimulation, positive comments and five star reviews can enrich consumers’ multisensory experiences and provide some support to their decision-making, who may want to know whether the product is good.

Fifth, arousal mediates the effect of visual perception (*H4*a), haptic imagery (*H4*b), and auditory stimulation (*H4*c) on purchase intent in e-customisation. The three hypotheses were supported with the results demonstrating that the influence of visual perception, haptic imagery, and auditory stimulation on purchase intent was mediated by perceived arousal in e-customisation. This is in line with the previous findings of Bakker et al. (2014) and Yang et al. (2020). Product e-customisation can trigger consumers’ pleasure and further influence their decision making. This study suggests that managers should consider both visual and auditory stimulation when designing e-customisation systems as it is necessary to explore more effective stimulus and arouse consumers’ positive emotions, when they are selecting and adjusting products online. Brand managers should not always focus on how to improve purchase intent and should instead conduct perception and emotion research in order to determine the usefulness of matching, adjusting, selecting and interacting functions in e-customisation systems. Fabric and haptic information can be conveyed *via* images and information that contributes to the recalling of the consumers’ previous experiences and may influence their emotions. For online retailers, this indicates that the aesthetics of online websites and clothing adjustment functions are more important than verbal description as far as increasing the pleasure of their consumers during their e-customisation experiences is concerned.

Sixth, perceived dominance mediates the effect of haptic imagery (*H5*c) on purchase intent in e-customisation. This result is in line with the previous findings of Constantinides (2004), Eroglu et al. (2001), Petit et al. (2019), and Zheng and Bensebaa (2021). This finding also contributes to the literature by providing evidence of the importance of how consumers perceive matching and adjustment. Our findings demonstrate that dominance mediates the relationship between perception and intent. In the fashion e-customisation environment, visual perception (*H5*a) and perceived auditory stimulation (*H5*b) were not significantly mediated by consumers’ perceived dominance, neither of them is not supported. As dominance was found to mediate the relationship between haptic mental imagery and purchase intent, retailers should consider consumers’ characteristics and preferences, then recommend personalized imagery information to them. For retailers, the fashion brands’ clothing interface will encourage haptic imagery cues during the online shopping experience, which could increase the latter’s purchase intent and their likelihood of purchasing online. Taken together, our findings suggest that visual perception, auditory stimulation, and haptic imagery have significant effects on arousal and purchase intent. These findings contribute to the study of sensory marketing and extend its effects to the field of fashion e-customisation. In addition, both visual perception and haptic imagery have significant effects on dominance. However, dominance was not affected by auditory stimulation. One possible explanation for this is that consumers might have found it inconvenient to use audio tools as the environment they were in may have been too noisy, or they may not have taken earphones with them. Another possibility is that consumers may be affected by influential environmental factors such as, for example, background noise deleteriously affecting their attention. It is clear that multisensory stimulation plays an important role in decision-making. In the context of e-customisation, arousal mediates the relationship between multisensory perception and purchase intent. Thus, it is necessary to improve the multisensory stimulation in the design of websites so as to appeal to consumers’ and help to maintain their positive emotion in order to facilitate their purchase of customised fashion products online.

### 5.1. Limitations and further research

There are some limitations that should be considered when exploring the relationships between factors (e.g., participants having negative emotions) in fashion e-customisation and purchase behaviour in future research. E-customisation is associated with technology development and marketing strategy, meaning that there are still opportunities to conduct further research. For example, 3D format images of clothing (e.g., a jacket) can stimulate vivid mental imagery and create a positive effect on purchase intent (Choi and Taylor, 2014). One potentially important topic for further research relates to possible gender differences in multisensory perception and purchase intent in fashion e-customisation and how these may contribute to online shopping behaviour. The reasons for the mediating effect of perceived dominance should be explored in future research. Lastly, as sensory marketing has developed over the years, there is still space to explore the sense with digital technology, virtual reality, and argument reality technology. How modern advanced technologies are adopted to stimulate human multisensory perception and the intention to purchase also needs more research.

## 6. Conclusion

The perceived usefulness of a website is significantly related to consumers’ positive attitude towards online clothing customisation (Cho and Fiorito, 2009). To explore the factors influencing a customer’s purchase intent in fashion e-customisation, we developed a conceptual model and explored the relationship between multisensory perception, positive emotions (perceived arousal and perceived dominance), and purchase intent. The mediating roles of perceived arousal and perceived dominance are also studied using a survey in China that should be explored among individuals with cultural differences. The findings highlight the existence of a relationship between visual perception, perceived auditory stimulation, haptic imagery, perceived arousal, perceived dominance, and purchase intent. First, the findings suggest that in order to encourage consumers’ purchase intent in e-customisation, retailers should work to improve the multisensory stimulation that they deliver. For example, the interface of the fashion e-customisation system should have unique functionality, or else consumers should be allowed to select the music following their preferences. In the sound virtual trial condition, consumers are willing to pay more for the jacket than when trying the item on in silence (Ho et al., 2013). The vocal sounds condition may stimulate consumers’ purchase behaviour in online shopping. Meanwhile, retailers should improve the interface design quality of and try to ensure that their website triggers the appropriate haptic metnal imagery create so as to increase their consumers’ arousal, which also affect purchase intent. According to different features, clothing categories and types can be divided. Detailed components of clothing and wearing occasions can be provided to consumers on the interface so as to support consumers purchase behaviour. Additionally, if brand managers seek to maintain their consumers’ purchase intent, developing the appropriate multisensory strategy represents a good approach, as the research outlined here demonstrates that multisensory perception can have significant effects on purchase intent. The mediating effect of perceived arousal also influences consumers’ decision-making.

Finally, the conclusions indicate that, in the context of fashion e-customisation, multisensory perception has a strong relationship with purchase intent for consumers. In fact, according to Spence et al. (2014) and Li et al. (2020), multisensory stimuli may exert a significant effect on consumers’ decision-making. Thus, looking to the future, online retailers will likely be able to benefit from multisensory approaches to e-customisation. For example, future research could contemplate the perception of taste and smell in the conceptual model, as the variable could provide a better explanation for consumers’ emotions and purchase intent.

## Data availability statement

The raw data supporting the conclusions of this article will be made available by the authors, without undue reservation.

## Ethics statement

Ethical review and approval was not required for the study on human participants in accordance with the local legislation and institutional requirements. Written informed consent for participation was not required for this study in accordance with the national legislation and the institutional requirements.

## Author contributions

PL contributed to design of the study. XG and CW contributed to the data collection. PL, XG, and CW performed the data analysis. PL wrote the first draft of the manuscript. PL and CS contributed to the manuscript revision, as well as reading and approving the submitted version.

## Funding

This work was supported by the Municipal Key Courses Program of Colleges and Universities in Shanghai (grant nos. 107-03-0007151, 107-03-0007402 and 107-03-0007092), the Industry-University Cooperation and Collaborative Education Project from the Ministry of Education in China (grant no. 202102122003).

## Conflict of interest

The authors declare that the research was conducted in the absence of any commercial or financial relationships that could be construed as a potential conflict of interest.

## Publisher’s note

All claims expressed in this article are solely those of the authors and do not necessarily represent those of their affiliated organizations, or those of the publisher, the editors and the reviewers. Any product that may be evaluated in this article, or claim that may be made by its manufacturer, is not guaranteed or endorsed by the publisher.

## Figures and Tables

**Table:** 

Construct	Item and measure	Sources
Visual perception (VIPE)	*Please indicate your level of agreement with the following statements about the e-customisation (1 = strongly disagree, 5 = strongly agree)*	Webster and Macleod (1988), Vieira (2010), Jang et al. (2018)
	VIPE1. Perceived colour matching	
	VIPE2. Perceived visual aesthetic	
	VIPE3. Perceived form matching	
Haptic imagery (HAIM)	*Please indicate your level of agreement with the following statements about the e-customisation (1 = strongly disagree, 5 = strongly agree)*	Longo et al. (2008), Witmer et al. (2005)
	HAIM1. I can freely adjust the product size on the screen	
	HAIM2. I can freely adjust the product style on the screen	
	HAIM3. I feel like I am trying on the products on the screen	
	HAIM4. By interacting with the system, I can easily determine the appearance of the products on me, such as by clicking on a button to select size, style, or taking a photograph	
Perceived auditory stimulation (PADS)	*Please indicate your level of agreement with the following statements about the e-customisation (1 = strongly disagree, 5 = strongly agree)*	Demoulin (2011), Dube and Morin (2001)
	PADS1. Perceived music tempo	
	PADS2. Perceived music pitch	
	PADS3. Perceived music tonality	
Perceived arousal (PARS)	*Please indicate your level of agreement with the following statements about the e-customisation*	Yang et al. (2020)
	PARS1. Bored-stimulated (*1 = bored, 5 = stimulated*)	
	PARS2. Dull-bright (*1 = dull, 5 = bright*)	
	PARS3. Calm-excited (*1 = calm, 5 = excited*)	
Perceived dominance (PDMN)	*Please indicate your level of agreement with the following statements about the e-customisation*	Yang et al. (2020)
	PDMN1. Influenced-influential (*1 = influenced, 5 = influential*)	
	PDMN2. Submissive-dominant (*1 = submissive, 5 = dominant*)	
	PDMN3. Guided-Autonomous (*1 = guided, 5 = autonomous*)	
Purchase intent (PIN)	*Please indicate your level of agreement with the following statements about the e-customisation (1 = strongly disagree, 5 = strongly agree)*	Pavlou (2003)
	PIN1. If the opportunity arises, I intend to buy an e-customised clothing from online stores.	
	PIN2. If given the chance, I can predict what I should buy from an online e-customised store in the future.	
	PIN3. I am likely to buy an e-customised clothing in an online store soon.	

## Appendix A

Measurements.
